# Perioperative transfusion in cardiovascular surgery and acute kidney injury

**DOI:** 10.1186/2197-425X-3-S1-A109

**Published:** 2015-10-01

**Authors:** G Gomez, E Curiel, MD Fernandez

**Affiliations:** Regional Hospital Málaga, Malaga, Spain

## Introduction

Controversy exists regarding the relationship of development of renal failure associated with blood transfusion in postoperative cardiac surgery.

## Objectives

Analyze the relationship between blood transfusion and quantity with the development of AKI in patients undergoing postoperative CCV.

## Methods

We analyzed patients undergoing CCV in 13 hospitals in the Andalusian community ARIAM (south Spain) collected in the CVS registry from the years 2008-2013.

We collected demographic and clinical variables surgery and transfusion requirements in the operating room during surgery. AKI defined as doubling of baseline creatinine elevation or above 2 mg / dl or the need Renal Replacement Therapy.

Qualitative variables were expressed as percentages and quantitative variables as mean and standard deviation. They have been used t-student and Chi 2 for the univariate analysis as required and binary logistic regression for multivariate analysis. Has employed a maximum of 5% alpha error.

## Results

Of the 7276 patients operated on at that time, we used for our analysis 2986 patients, of which we had all the information regarding perioperative blood transfusion.

A total of 817 cases (27.4%) have developed renal dysfunction. 918 patients (30´7%) have not received transfusion and the remaining 2068 (69´3%) did it, in which:

1 RCT 339 (11´4%), 2 RCT 501 (16.8%), >2 RCT 530 (17.7%), 651 platelets (21.8%), plasma 440 (14.7%).

We found a relationship between transfusion of any blood (red cells, plasma and platelets) with the development of AKI (p < 0.05). In the case of red blood cells and plasma, the relationship is stronger as we increase the number of transfusions.

In multivariate analysis, predictors of renal failure were EC time, the EuroSCORE and RBC transfusion only.

## Conclusions

In our study we found that perioperative blood transfusion is directly related to the development of AKI, with greater force as the number of units transfused increases. Transfusion of RC was independent predictor of AKI.

**Figure 1:**
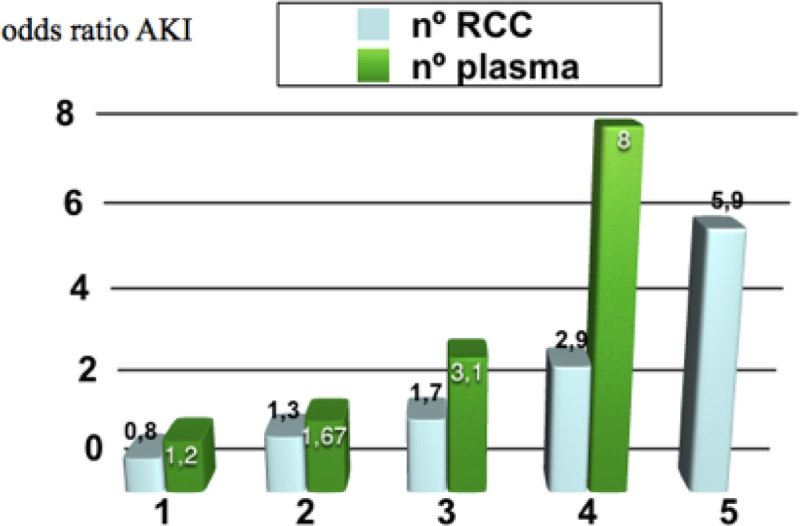
**aki transfuse.**

